# Palmitic Amide Triggers Virus Life Cycle *via* Enhancing Host Energy Metabolism

**DOI:** 10.3389/fmicb.2022.924533

**Published:** 2022-06-09

**Authors:** Xinyi Zhang, Jianjian Zhuang, Liquan Huang, Xiaobo Zhang

**Affiliations:** College of Life Sciences, Laboratory for Marine Biology and Biotechnology of Pilot National Laboratory for Marine Science and Technology (Qingdao) and Southern Marine Science and Engineering Guangdong Laboratory (Zhuhai), Zhejiang University, Hangzhou, China

**Keywords:** bacteriophage, virus life cycle, palmitic amide, acetate kinase, energy metabolism

## Abstract

Viruses contribute to the mortality of organisms, consequentially altering biological species composition of an ecosystem and having a threat on human health. As the most famous model for the initiation of virus infection, the Hershey-Chase experiment has revealed that on infection, the bacteriophage genomic DNA is injected into its host bacterium, while the viral capsid is left on the outer membrane of host cell. However, little is known about the injection of any other materials into the cytoplasm of host cells along with genomic DNA to trigger the virus life cycle. In this study, the results showed that palmitic amide packaged in the virions of GVE2, a bacteriophage infecting deep-sea hydrothermal vent thermophile *Geobacillus* sp. E263, promoted virus infection. Palmitic amide was interacted with acetate kinase to increase its enzymatic activity, thus enhancing the acetate-mediated energy metabolism. Furthermore, palmitic amide promoted tricarboxylic acid cycle (TCA cycle) to support virus infection. These data indicated that palmitic amide, packaged in the virions, might serve as a second messenger at the initiation step of virus infection by enhancing the host energy metabolism. Therefore our study revealed a novel mechanism for the initiation of the virus life cycle.

## Introduction

Being more than 10^31^ in number, viruses are the most abundant biological entities on the earth (Call et al., 2021). The viruses have significant impacts on the global biogeochemical cycles and human health (Wei and Zhang, 2010; He et al., 2017; Call et al., 2021), thus attracting more and more attentions in recent years. The viruses can also serve as vehicles to transfer genes. It is estimated that 10^24^ genes are shuttled from viruses to hosts each year, which indicates that the viruses may be a major source of genetic creativity (Dennehy, 2014). The knowledge about the process of virus infection is first obtained from bacteriophages (phages) (Sharma et al., 2017; Schmalstig et al., 2021). During the phage infection, the first step for phage infection is the interaction between phages and the receptors on its host cells (Schmalstig et al., 2021). The tail protein of phages would degrade the cell wall of bacteria and the genomic nucleotide acids of phages are injected into the host cells (Schmalstig et al., 2021). The subsequent replication strategy of phage can be classified into virulent or template phage (Howard-Varona et al., 2017). In the lytic cycle of phages, the phages utilize the machinery of hosts to complete the replication of viral genomes and the assembly of virions (Schmalstig et al., 2021). At present, it has been well characterized that the infecting phage must transmit its genome out of the viral particle and into the cytoplasm of host cell (Weaver, 2012; Cumby et al., 2015; Schmalstig et al., 2021). However, it remains unknown whether any other components, such as metabolites, are packaged in the virions and transmitted into the host cells along with the viral genomes to initiate the virus life cycle.

In recent years, it is found that metabolites, especially compounds, play very important roles in virus infection (Kronheim et al., 2018). Three naturally produced small molecules including daunorubicin, doxorubicin and acriflavine can inhibit phage infection in *Streptomyces* by inserting them into the genomic DNA of phages, leading to the blocking of phage DNA circularization or interaction with the proteins that occur after DNA injection into host cells but before DNA replication (Kronheim et al., 2018). In a deep-sea hydrothermal vent bacterium *Geobacillus* sp. E263, the compound tryptophol, which is elevated in the host bacteria in response to the phage GVE2 infection, suppresses the infection of GVE2 by interacting with the host’s Clp protease (Jin et al., 2015). Except for phages, secondary metabolites take great effects on the infection of eukaryotic viruses (Son et al., 2013; Xu et al., 2016). As reported, a compound, named emricasan that is a pan-caspase inhibitor, can inhibit Zika virus infection by inhibiting Zika-induced increases of caspase-3 activity (Xu et al., 2016). For human herpesvirus (HSV), its infection is suppressed by glycyrrhizic acid that is isolated from licorice (Son et al., 2013). The compound glycyrrhizic stimulates the host cells’ immune responses to disrupt HSV infection. Although small molecular compounds have been found to play very important roles in the process of virus infection, whether some compounds can be packaged in the virions and released into the host cells to trigger virus infection at the initial infection stage has not been explored.

To explore the underlying mechanism for the initiation of virus life cycle, the compounds packaged in the virions of GVE2, a bacteriophage infecting deep-sea hydrothermal vent thermophile *Geobacillus* sp. E263 (Wei and Zhang, 2010), were characterized in the present investigation. The results showed that the compound palmitic amide, packaged in the virions, functioned as a second messenger to trigger the virus life cycle in the cytoplasm of host cells by enhancing the host tricarboxylic acid cycle (TCA cycle) *via* the acetate pathway.

## Materials and Methods

### Virus Purification and Infection

Deep-sea thermophile *Geobacillus* sp. E263 (China General Microbiological Culture Collection Center accession no. CGMCC1.7046) was cultured in the TTMM medium (0.8% tryptone, 0.4% yeast extract, 0.2% NaCl and 0.2% MgSO_4_⋅7H_2_O) at 60°C. Thermophilic bacteriophage GVE2 was purified from GVE2-infected E263 using CsCl gradient centrifugation. Briefly, the virus-infected E263 was cultured overnight and then treated with mitomycin C (1 μg/mL) for 2 h, followed by culture in TTMM medium at a ratio of 1:100 for 6 h. The GVE2-infected bacteria were incubated with DNase and RNase for 1 h at room temperature. Subsequently the bacteria were incubated with 1M NaCl on ice for 1 h. After centrifugation at 6,500 × g for 30 min, the supernatant was added with PEG6000 (polyethylene glycol 6000) at a final concentration of 10% (m/v) and the mixture was incubated at 4°C overnight. The virions were collected by centrifugation at 40,000 × g for 2 h. Subsequently the virions were resuspended in SM buffer (400 mM NaCl, 20 mM MgSO_4_7H_2_O, 50 mM Tris-HCl, pH7.5) to facilitate virus infection, followed by CsCl gradient centrifugation at 200,000 × g for 3 h. The virion band was collected and desalted by dialysis against SM buffer. The purity of GVE2 virions was examined using transmission electron microscopy.

At logarithmic phase, the strain E263 was infected with its bacteriophage GVE2 at a multiplicity of infection (MOI) of 0.5. After 10 min adsorption, the unadsorbed phage was removed by centrifugation at 300 × g. The pellet was resuspended in fresh TTMM medium and then cultured at 60°C. An aliquot of 50 μl of the infected bacteria was collected every 30 min. The GVE2 copies were quantified by quantitative real-time PCR using GVE2-specific primers (5′-ATCGGTTGTACTAACTTAAC-3′ and 5′-GCTTGTCGTATTCCTTATC-3′) and TaqMan probe (5′-FAM-CCGTCTTGTTCGTTGTCTCTGC-Eclipse-3′).

### Extraction and Identification of Compounds

The GVE2 virions (1 × 10^9^) were incubated with 80% methanol at 4°C overnight to extract the compounds packaged in the virions. The compounds of GVE2-infected *Geobacillus* sp. E263 at 24 h post-infection were also extracted. After centrifugation at 12,000 × g for 1 h, the pellet was resolved in 80% methanol. The extraction procedure was repeated for three times. Subsequently the supernatants were combined and dried under vacuum at 50°C.

For the identification of compounds using gas chromatography coupled tandem mass spectrometry (GC-MS), the extracted compounds were subjected to oximation and silylation derivatization. Briefly, the dried compounds were reacted with 20 μl of 20 mg/mL methoxyamine-HCl in pyridine (Sigma, United States) at 35°C for 125 min, followed by silylation using 20 μl of N,O-bis (trimethylsilyl) trifluoroacetamide and 1% trimethylchlorosilane (Sigma, United States) at 35°C for 60 min. Then the compounds were analyzed by GC-MS using a DSQ II Quadrupoles mass spectrometry (Thermo Electron Corporation, United States). The mass spectrometry was operated at electron impact mode at 70 eV. The source temperature was set at 200°C. To identify the compounds, the GC-MS data of each peak was searched against NIST (National Institute of Standards and Technology, United States) library using MassHunter software (Agilent Technology, United States).

For the identification of compounds using liquid chromatography coupled tandem mass spectrometry (LC-MS), the extracted compounds were analyzed by a Dionex U3000 UHPLC system (Thermo Fisher Scientific, Waltham, United States) fitted with a Q-Exactive quadrupole-Orbitrap mass spectrometer (Thermo Fisher Scientific, Waltham, United States). Progenesis QI v2.3 software was applied to identify the compounds with the basis of public databases including the human metabolome database (HMDB), lipidmaps (v2.3) and metlin database.

To confirm the identified compounds, the standard compounds were purchased from Aladdin Chemicals (Shanghai, China) and dissolved in 80% methanol. The standard compounds were analyzed by GC-MS or LC-MS. The mass spectral date of each peak were compared with those of the samples.

### One-Step Growth Curve of GVE2

*Geobacillus* sp. E263 cells were suspended in fresh TTMM medium and then incubated with GVE2 at a MOI of 0.1 for 30 min at room temperature. The bacteria were centrifuged at 5,000 × g for 10 min, followed by resuspension with fresh TTMM medium to terminate the GVE2 adsorption. The host cells were grown at 60°C and the supernatant of E263 culture was collected every hour. The bacterial supernatant was subjected to quantitative real-time PCR to quantify GVE2 copies. The experiments were biologically repeated three times.

### Influence of Metabolites Packaged in GVE2 Virions on Phage Infection

*Geobacillus* sp. E263 was infected with GVE2 and then valine, threonine, proline, glutamic acid, thiodiacetic acid, ribose, isobutene glycol or palmitic amide at 100μM was added into the GVE2-infected E263. Palmitic amide was dissolved in 0.2% dimethyl sulfoxide (DMSO), while the remaining compounds were dissolved in phosphate buffered saline (PBS). As a control, DMSO or PBS was included in the assays. At different time post-infection, the GVE2 copies were examined.

### Transcriptomic Analysis by RNA-Seq

*Geobacillus* sp. E263 was added with dimethyl sulfoxide (DMSO) or palmitic amide, followed by culture in TTMM medium at 60°C for 24 h. After centrifugation at 5,000 × g for 10 min, the bacterial pellet was subjected to RNA extraction using TRIzol reagent (Invitrogen, United States). Then the extracted RNA was sequenced by Major Biotechnology Co., Ltd (Shanghai, China) with Illumina HiSeq 2500 platform. The raw sequence reads were processed to clean reads by SeqPrep^1^ and Sickle (Version 1.33).^2^ The clean reads were mapped into the genome of *Geobacillus* sp. E263 (GenBank accession no CP041632) with the program Bowtie.^3^ The gene expression quantification was analyzed by RSEM software.^4^ The pathway enrichment analysis of differential expressed genes was based on the KEGG (Kyoto encyclopedia of genes and genomes) database.

### Detection of Gene Expression by Quantitative Real-Time PCR

To examine the expression profiles of the differentially expressed genes, total RNAs were extracted from *Geobacillus* sp. E263 using TransZol Up Plus RNA kit (TransGen Biotech, Beijing, China), followed by the synthesis of cDNA with HiScript II Q RT SuperMix for qPCR kit (+ gDNA wiper) (Vazyme, Nanjing, China). Quantitative real-time PCR was performed with gene-specific primers (universal stress protein, 5′-TGCTATTGATGGTTCCAAAG-3′ and 5′-GCTCCGTAAATCAATGACG-3′; ribosome-associated translation inhibitor RaiA, 5′-TTTGAGATTGTCCGCACG-3′ and 5′-ACACGATGTTTGTGCGGT-3′; DoxX family protein, 5′-GGATTTGACGCAACAGGA-3′ and 5′- CACTA ACACATTGAACACCTCG-3′; alcohol dehydrogenase AdhP, 5′-GCACCTGCCGATTATGTC-3′ and 5′-TCCGTAAATGGCTA CCCA-3′; 30S ribosomal protein S4, 5′-TGGTCCAAGTCA ACGCAG-3′ and 5′-CCGTGTTTACCAGGCATT-3′). The Hieff qPCR SYBR Green Master Mix (Yeasen Biotechnology, Shanghai, China) was used in quantitative real-time PCR.

### Gene Ontology and Kyoto Encyclopedia of Genes and Genomes Analysis

The Gene Ontology (GO) analysis was performed based on the GO database^5^ using blast.^6^ The KEGG analysis was carried out using the Kyoto Encyclopedia of Genes and Genomes (KEGG) database (Version 2017.08).^7^

### Identification of the Proteins Bound to Palmitic Amide

To obtain the proteins bound to palmitic amide, palmitic amide was coupled with NHS-Activated Beads 4FF (Smart-Lifesciences, Changzhou, China). Before use, the beads were washed with 1 mM HCl for three times and then balanced with coupling buffer (0.2M NaHCO_3_, 0.1M NaCl, pH8.0). The beads were incubated with 1 mM palmitic amide dissolved in DMSO or DMSO at room temperature overnight. After rinse for five times, the beads were blocked with blocking buffer (0.1M Tris-HCl, pH8.5) at room temperature for 1.5 h. The beads were washed with washing buffer 1 (0.1M CH_3_COONa-CH_3_COOH, 0.5M NaCl, pH3.0), washing buffer 2 (0.1M Tris-HCl, 0.5M NaCl, pH8.0) and distilled water for two times, respectively. Subsequently the beads were balanced with PBS. The proteins extracted from *Geobacillus* sp. E263 were incubated with the palmitic amide-coupled beads at 4°C overnight. The proteins bound to palmitic amide were analyzed by SDS-PAGE with Coomassie brilliant blue staining. The proteins were identified by mass spectrometry.

### Isothermal Titration Calorimetry

Isothermal titration calorimetry (ITC) was conducted using MicroCal PEAQ-ITC Instrument (Malvern Panalytical, Northampton, MA, United States) at 25°C. The protein (20 μM) was added with the compound (200 μM) at the rate of 2 μl/120s. The results were analyzed by the MicroCal PEAQ-ITC Analysis Software. The dissociation constant (Kd) and enthalpy change (ΔH) were calculated by the software directly.

### Impact of Palmitic Amide on Acetate Kinase Activity

The enzymatic activity of acetate kinase was examined using the universal kinase activity kit (R&D Systems, Minneapolis, United States). Briefly palmitic amide at different concentrations (10 and 100 μM) was incubated with the purified recombinant acetate kinase (100 μg/mL) in the assay buffer (R&D Systems) at 60°C for 10 min, followed by the addition of phosphatase (R&D Systems) and then incubation at room temperature for 10 min. As a control, DMSO was included in the assays. Subsequently Malachite Green Reagent A (R&D Systems) was added into the mixture to terminate the reactions. After incubation with Malachite Green Reagent B (R&D Systems) for 20 min at room temperature to stabilize the color development, the optical density of each reaction was detected at 620 nm. The acetate kinase activity was calculated according to the standard curve.

### Detection of Bacterial Citrate Synthase Activity

*Geobacillus* sp. E263 was added with different concentrations of sodium acetate (500 μM, 1 mM, and 10 mM) or palmitic amide (10 and 100 μM) and then cultured at 60°C for 24 h. The citrate synthase activity was detected using the citrate synthase activity detection kit (Beijing Solarbio Science and Technology, Beijing, China) according to the manufacturer’s instructions.

### Statistical Analysis

One-way analysis of variance was used to calculate the mean and standard deviations of three replicates. The statistical significance of differences between different treatments were analyzed using Student’s *t*-test.

## Results

### Identification of Metabolites Packaged in Virus Particles

**FIGURE 1:**
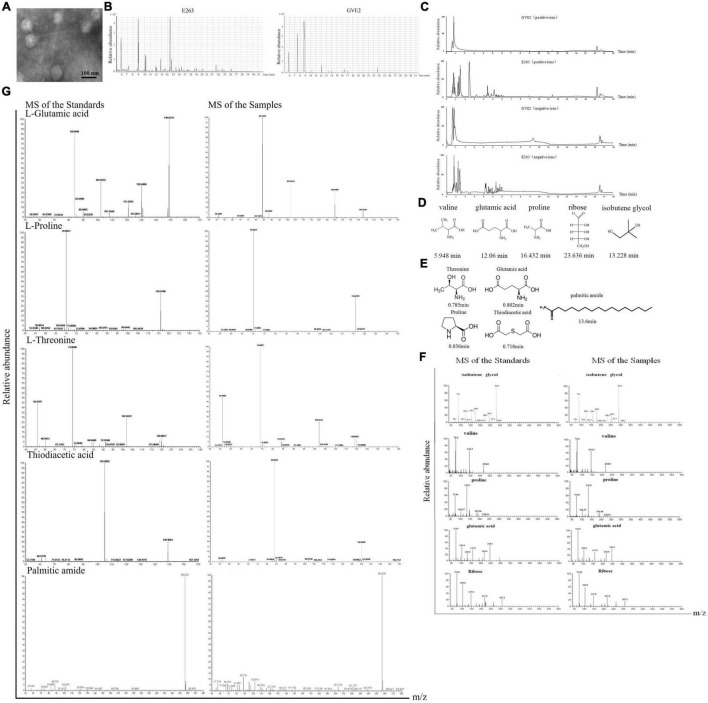
Identification of metabolites packaged in virus particles. **(A)** Purification of virions. The purity of purified GVE2 virions was examined with transmission electron microscopy. Scale bar, 100 nm. **(B)** GC-MS analysis of the metabolites extracted from the purified virions and the virus-infected bacteria. *Geobacillus* sp. E263 was infected by its bacteriophage GVE2. At 1 h post-infection, the bacteria were subjected to compound extraction. The compounds extracted from the purified GVE2 virions and the GVE2-infected E263 were analyzed by GC-MS. Relative abundance of the compounds was plotted with retention time in minute. **(C)** LC-MS analysis of the metabolites packaged in GVE2 virions. The compounds extracted from the purified GVE2 virions and the virus-infected bacteria were identified by LC-MS. **(D)** The compounds of GVE2 virions identified by GC-MS. The retention time was indicated in minutes. **(E)** The compounds of GVE2 virions identified by LC-MS. **(F)** The GC-MS analysis of the standards and the samples (the compounds from the purified GVE2 virions). The retention time was indicated in minutes. **(G)** The LC-MS analysis of the standards and the samples (the compounds from the purified GVE2 virions).

To explore the initiation of bacteriophage infection in host, a thermophilic bacteriophage GVE2, purified from GVE2-infected *Geobacillus* sp. E263 of deep-sea hydrothermal vent (Liu and Zhang, 2008), was characterized. The TEM results indicated that the thermophilic bacteriophage GVE2 particles were purified from its host *Geobacillus* sp. E263 using CsCl gradient centrifugation (Figure 1A). Then the metabolites from GVE2 virions and GVE2-infected E263 were extracted. The results of GC-MS and LC-MS revealed that the metabolite peaks of GVE2 virions were matched to those of GVE2-infected E263 (Figures 1B,C), showing that the metabolites packaged in GVE2 virions were obtained from its host bacteria. To identify the metabolites packaged in GVE2 virions, the GC-MS peaks were searched against NIST (National Institute of Standards and Technology) library. In total, seven compounds were obtained. Among them, two compounds were the components of the solution (Tris-HCl, retention time at 8.9 min; glycerol, retention time at 7.5 min) and the remaining compounds were identified to be three amino acids (proline, valine and glutamic acid), one saccharide (ribose) and isobutene glycol (Figure 1D). Based on LC-MS analysis by searching against HMDB, lipidmaps (v2.3) and metlin database, the compounds packaged in GVE2 virions were identified to be threonine, glutamic acid, proline, thiodiacetic acid and palmitic amide (Figure 1E).

To confirm the GC-MS and LC-MS data, the standards of the identified compounds were analyzed by GC-MS and LC-MS, respectively. The results of GC-MS showed that the standards and the samples (the compounds extracted from the purified GVE2 virions) shared the same mass spectra profiles (Figure 1F), thus confirming the GC-MS data. For the LC-MS data, threonine, glutamic acid, proline and palmitic amide were confirmed (Figure 1G). But the LC-MS data of the sample was not matched with that of the standard thiodiacetic acid (Figure 1G).

Taken together, the findings revealed that proline, valine, glutamic acid, threonine, ribose, isobutene glycol and palmitic amide could be packaged in the GVE2 virions purified using CsCl gradient centrifugation.

### Roles of Metabolites Packaged in Viral Particles on Phage Replication

**FIGURE 2:**
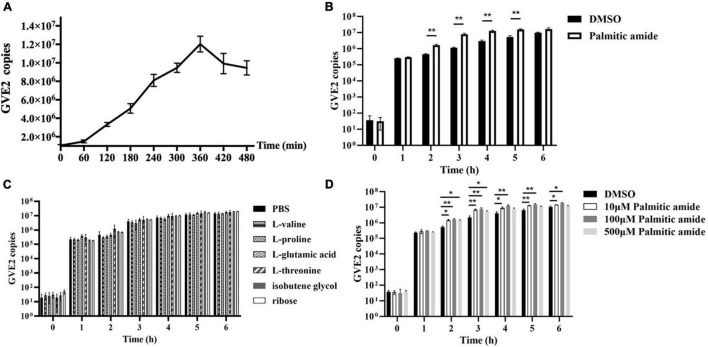
Roles of metabolites packaged in viral particles on phage replication. **(A)** One-step growth curve of GVE2 in its host *Geobacillus* sp. E263. **(B)** Influence of palmitic amide on GVE2 replication. The GVE2-infected *Geobacillus* sp. E263 was incubated with palmitic amide which was dissolved in DMSO. DMSO alone was used as a control. At different time post-infection, the GVE2 copy was measured by quantitative real-time PCR. **(C)** Effects of valine, proline, glutamic acid, threonine, ribose and isobutene glycol on phage replication. The GVE2-infected *Geobacillus* sp. E263 was added with valine, proline, glutamic acid, threonine, ribose or isobutene glycol dissolved in PBS. PBS alone was used as a control. At different time post-infection, the GVE2 copy was examined. **(D)** Influence of palmitic amide at different concentrations on GVE2 replication. The GVE2-infected *Geobacillus* sp. E263 was added with palmitic amide at 10, 100, or 500 μM, followed by the detection of GVE2 copies with quantitative real-time PCR. DMSO was used as a control. In all panels, the statistical significance between treatments were indicated with asterisks (*p < 0.05; **p < 0.01).

To explore the roles of metabolites packaged in viral particles on phage replication, one of four amino acids (valine, threonine, proline, and glutamic acid), ribose, isobutene glycol or palmitic amide was mixed with GVE2 and then incubated with the host *Geobacillus* sp. E263 cells, followed by the evaluation of GVE2 infection. The results of one-step growth curve of GVE2 indicated that GVE2 reached the maximum copies at 6 h post-infection (Figure 2A).

When palmitic amide was added into the GVE2-infected *Geobacillus* sp. E263, the GVE2 copy was significantly increased compared with the control (DMSO) (Figure 2B). However, valine, proline, glutamic acid, threonine, ribose and isobutene glycol had no effect on the GVE2 replication (Figure 2C). These data demonstrated that palmitic amide packaged in virus particles could promote phage replication.

To evaluate the influence of palmitic amide at different concentrations on GVE2 replication, the GVE2-infected *Geobacillus* sp. E263 was added with three different concentrations of palmitic amide (10, 100, and 500 μM). The results indicated that palmitic amide at 100 μM presented the most significant promotion of GVE2 replication (Figure 2D).

Collectively, these findings revealed that palmitic amide packaged in GVE2 virions promoted phage replication in host cells.

### Pathways Mediated by Palmitic Amide Packaged in Viral Particles

**FIGURE 3:**
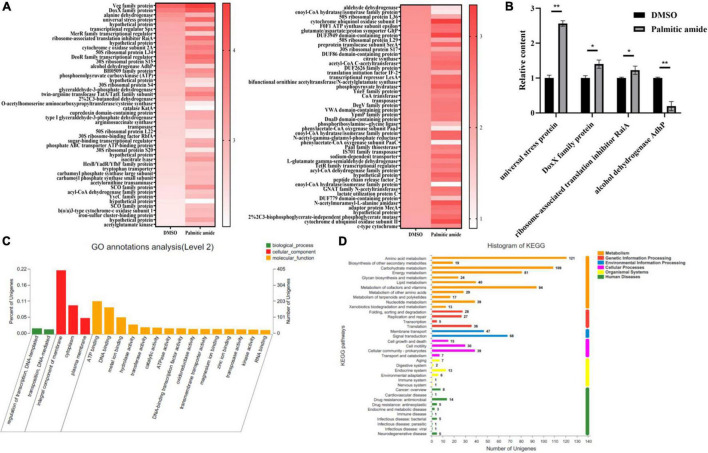
Pathways mediated by palmitic amide packaged in viral particles. **(A)** Heat map of the differentially expressed genes in *Geobacillus* sp. E263 treated with palmitic amide or DMSO. *Geobacillus* sp. E263 was added with palmitic amide or DMSO and then cultured for 24 h. Subsequently the mRNAs of *Geobacillus* sp. E263 were subjected to sequencing. **(B)** Confirmation of the differentially expressed genes. *Geobacillus* sp. E263 was treated with DMSO or palmitic amide, followed by examination with quantitative real-time PCR. Four genes were randomly selected for this analysis. The statistical significance of difference between treatments was indicated with asterisks (*p < 0.05; **p < 0.01). **(C)** GO analysis of differentially expressed genes between *Geobacillus* sp. E263 treated with palmitic amide and DMSO. **(D)** KEGG analysis of differentially expressed genes between *Geobacillus* sp. E263 trated with palmitic amide and DMSO.

To reveal the pathways mediated by palmitic amide packaged in viral particles, RNA-seq was performed using the palmitic amide-challenged *Geobacillus* sp. E263 (GenBank accession number PRJNA833234). The results showed that 73 genes were significantly upregulated and 23 genes were significantly downregulated in the palmitic amide-challenged *Geobacillus* sp. E263 compared with the control (Figure 3A). To confirm the RNA-seq data, 4 genes were randomly selected to examine their expression profiles in the palmitic amide-challenged *Geobacillus* sp. E263. The quantitative real-time PCR results were consistent with the RNA-seq data (Figure 3B), confirming the data of transcriptome analysis.

To identify the categories of the differentially expressed genes between the bacteria treated with palmitic amide and DMSO, GO and KEGG pathway analyses were performed. The results indicated that most of the differentially expressed genes of palmitic amide-challenged *Geobacillus* sp. E263 were involved in integral component of membrane and ATP binding (Figure 3C), suggesting that palmitic amide played important roles in bacterial energy metabolism. The KEGG pathway analysis showed that the differentially expressed genes of palmitic amide-challenged bacteria were clustered into 37 pathways (Figure 3D). Most of pathways were associated with metabolisms, including carbohydrate metabolism, energy metabolism and amino acid metabolism (Figure 3D). Moreover, some genes of tricarboxylic acid cycle (TCA cycle), such as citrate synthase, isocitrate dehydrogenase, succinate dehydrogenase and malate dehydrogenase, were significantly upregulated in palmitic amide-challenged *Geobacillus* sp. E263. These data suggested that palmitic amide was involved in host energy metabolism.

Taken together, these data demonstrated that palmitic amide packaged in viral particles was associated with host energy metabolism.

### The Proteins Interacted With Palmitic Amide

**FIGURE 4:**
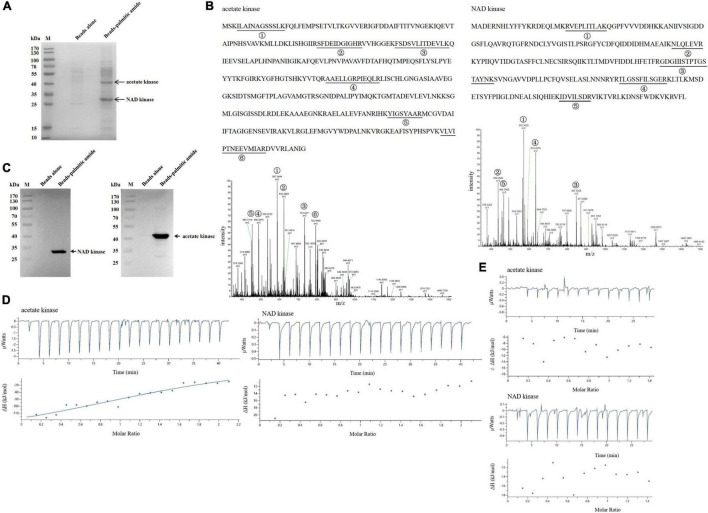
The proteins interacted with palmitic amide. **(A)** The proteins bound to palmitic amide. *Geobacillus* sp. E263 was lysed by ultrasonication and the proteins in supernatant were incubated with palmitic amide-coupled NHS-activated beads. The proteins were separated by SDS-PAGE with Coomassie brilliant blue staining. NHS- activated beads alone was used as a control. The arrows represented the differential proteins. M, protein marker. **(B)** Identification of the proteins bound to palmitic amide by mass spectrometry. The proteins were identified to be acetate kinase and nicotinamideadenine dinucleotide (NAD) kinase, respectively. The matched peptides were indicated with underlines. **(C)** Western blot analysis of the proteins interacted with palmitic amide. The proteins extracted from *Geobacillus* sp. E263 were incubated with palmitic amide-coupled beads, and then subjected to Western blot using NAD kinase-specific or acetate kinase-specific antibody. The target protein was indicated with an arrow. M, protein marker. **(D)** Interaction between palmitic amide and acetate kinase or NAD kinase. The purified recombinant acetate kinase (20 μM) or NAD kinase (20 μM) was incubated with palmitic amide (200 μM) to conduct isothermal titration calorimetry (ITC). **(E)** Evaluation of the binding of palmitic acid to acetate kinase or NAD kinase by ITC. Palmitic acid (200 μM) was incubated with the purified recombinant acetate kinase (20 μM) or NAD kinase (20 μM) to perform ITC.

To explore the mechanism of palmitic amide in virus infection, the proteins bound to palmitic amide were characterized. The data of affinity chromatography using palmitic amide showed that two proteins were specifically bound to palmitic amide (Figure 4A). Based on mass spectrometric data, the two proteins were identified to be acetate kinase and nicotinamideadenine dinucleotide (NAD) kinase of *Geobacillus* sp. E263 (Figures 4A,B). Western blots further confirmed the mass spectrometric results (Figure 4C). These data indicated that palmitic amide might be interacted with NAD kinase and acetate kinase.

To further characterize the direct interaction between palmitic amide and acetate kinase or NAD kinase, isothermal titration calorimetry (ITC) analysis was performed. The results showed that the dissociation constant (Kd) of the binding of palmitic amide (200 μM) to acetate kinase (20 μM) was 13.2 ± 28.1 μM, while the Kd of palmitic amide to NAD kinase (20 μM) was not detected (Figure 4D), indicating that palmitic amide could directly bind to acetate kinase but not NAD kinase. To determine the active group of palmitic amide to interact with acetate kinase, ITC analysis was conducted to evaluate the interaction between palmitic acid and acetate kinase or NAD kinase. The results revealed that the Kd of the binding of palmitic acid to acetate kinase or NAD kinase was not detected (Figure 4E), showing that amide of palmitic amide was responsible for the interaction between palmitic amide and acetate kinase.

Taken together, these data indicated that palmitic amide was interacted with acetate kinase, suggesting that palmitic amide was associated with the acetate-mediated energy metabolism to promote virus infection.

### Influence of Palmitic Amide-Mediated Energy Metabolism on Virus Infection

**FIGURE 5:**
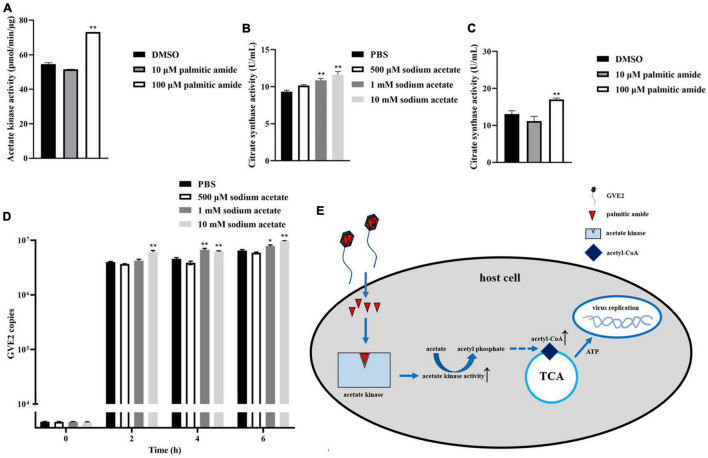
Influence of palmitic amide-mediated energy metabolism on virus infection. **(A)** Influence of palmitic amide on the activity of acetate kinase. The acetate kinase was incubated with palmitic amide at different concentrations. Then the enzymatic activity was examined. DMSO was included in the assays as a control. **(B)** Role of sodium acetate in tricarboxylic acid cycle (TCA cycle). *Geobacillus* sp. E263 was added with different concentrations of sodium acetate. Twenty four hours later, the bacterial citrate synthase activity was determined. PBS was used as a control. **(C)** Impact of palmitic amide on the citrate synthase activity. *Geobacillus* sp. E263 was added with 10 or 100 μM of palmitic amide and then cultured at 60°C for 24 h. DMSO was used a control. The bacterial citrate synthase activity was examined. **(D)** Effects of the palmitic amide-mediated TCA cycle on virus infection. The GVE2-infected *Geobacillus* sp. E263 was added with sodium acetate at different concentrations (500 μM, 1 mM, and 10 mM). At different time after treatment, the GVE2 copies were determined using quantitative real-time PCR. PBS was used as a control. **(E)** Model for the palmitic amide-mediated energy metabolism in virus infection. In all panels, the statistical significance of difference between treatments was indicated with asterisks (*p < 0.05, **p < 0.01).

To reveal the impact of palmitic amide-mediated energy metabolism on virus infection, acetate kinase was incubated with palmitic amide, followed by the examination of acetate kinase activity. The results showed that palmitic amide (100 μM) significantly enhanced the activity of acetate kinase compared with the control (Figure 5A), indicating that palmitic amide might be involved in TCA cycle *via* the acetate pathway. The addition of sodium acetate significantly increased the activity of citrate synthase, the key enzyme of TCA cycle (Figure 5B). Furthermore the results demonstrated that palmitic amide (100 μM) significantly enhanced the citrate synthase activity (Figure 5C), showing that palmitic amide promoted TCA cycle. These data presented that palmitic amide could promote the acetate-mediated TCA cycle.

To further evaluate the effects of palmitic amide-mediated TCA cycle on virus infection, sodium acetate was added into the GVE2-infected *Geobacillus* sp. E263, followed by the detection of GVE2 copies. The quantitative real-time PCR data reveled that the addition of sodium acetate significantly increased the GVE2 copies in *Geobacillus* sp. E263 (Figure 5D), showing that the enhancement of TCA cycle promoted virus infection.

Taken together, these findings demonstrated that when GVE2 infected host cells, palmitic amide, packaged in the virions, was released into the cytoplasm to increase the activity of acetate kinase, leading to the enhancement of TCA cycle, thus further supporting virus infection *via* the energy supply (Figure 5E).

## Discussion

Viruses can finish their life cycle only in host cells. The model for the initiation of virus life cycle comes from bacteriophages (phages) (Labrie et al., 2010; Schmalstig et al., 2021). In the process of phage infection, the phages first absorb on the surface of the host bacterial cells by interacting with the specific receptors. Subsequently the phage genomes packaged in the virions are injected into the host cells to initiate the virus life cycle and further to complete the replication of viral genomes and the assembly of virions using the host machinery (Labrie et al., 2010; Schmalstig et al., 2021). Obviously the viral genome alone is difficult for viruses to manipulate their host machinery to trigger virus life cycle. However, it remains poorly described whether there are some molecules from the virions entering into the host cells except for viral genomes to initiate the virus life cycle. In this study, the results revealed that some compounds, such as palmitic amide, could be packaged in the virions. When infecting the host cells, the viruses released the packaged palmitic amide into the host cytoplasm along with the viral genomes to manipulate the host energy metabolism (TCA cycle) to trigger the virus life cycle. At present, there is little information about the biological function of palmitic amide. Therefore our findings presented novel insights into the underlying mechanism of the initiation of virus life cycle. As described previously (Zhang et al., 2021), some compounds including palmitic amide can be packaged in the virions of white spot syndrome virus, a virus infecting eukaryotic crustaceans, and released into the host cytoplasm to trigger the virus life cycle *via* enhancing the host energy metabolism (glycolysis). In this context, the findings of this and previous studies revealed that palmitic amide was required for both prokaryotic and eukaryotic viruses to trigger the host energy machinery, thus initiating the virus life cycle.

In the present study, the results showed that in the initiation process of virus infection, the compound palmitic amide packaged in the virions of phages could be released into the host cells to increase the activity of acetate kinase and further to enhance the TCA cycle, indicating that palmitic amide could enhance the energy metabolism of host bacteria *via* the acetate pathway. In this study, the direct interaction between palmitic amide and acetate kinase was characterized using ITC that was performed at 25°C. Although acetate kinase was encoded by thermophile *Geobacillus* sp. E263, the ITC results showed that palmitic amide could interact with acetate kinase at room temperature. In the previous investigation (Zhang et al., 2021), the findings revealed that palmitic amide packaged in the virions of white spot syndrome virus (a eukaryotic virus) triggered the virus life cycle in the cytoplasm of eukaryotic host by enhancing host glycolysis through increasing the enzymatic activity of triosephosphate isomerase, a key enzyme of glycolysis. At the initial infection stage of both prokaryotic and eukaryotic viruses, palmitic amide, the intracellular produced non-protein micromolecule, could enhance the activity of the key enzymes of the host energy metabolism to trigger the signal transduction pathway. Therefore palmitic amide might be a second messenger packaged in the virions, which was responsible for the initiation of virus life cycle. Up to date, some second messengers have been identified (Endo, 2006; Wright et al., 2015; Friebe et al., 2020). Cyclic adenosine monophosphate (cAMP), a famous second messenger, performs its function by activating protein kinase A, exchange protein activated by cAMP or cyclic nucleotide gated channels (Wright et al., 2015). Cyclic guanosine monophosphate (cGMP) is a unique second messenger which regulates various physiological responses by driving the activation of cGMP-dependent protein kinases, ion channels or phosphodiesterases (Friebe et al., 2020). Ca^2+^ can also serve as a second messenger in the excitation-contraction coupling of skeletal muscle (Endo, 2006). In this context, our findings provided a potential second messenger packaged in the virions and presented novel insights into the underlying mechanism of the initiation of virus life cycle.

## Data Availability Statement

The data has been deposited in the NCBI database. The accession number is PRJNA833234.

## Author Contributions

XYZ and XBZ designed the study. XYZ and JZ performed the experiments. XYZ, LH, and XBZ wrote and edited the manuscript. All authors discussed the results and commented on the manuscript.

## Conflict of Interest

The authors declare that the research was conducted in the absence of any commercial or financial relationships that could be construed as a potential conflict of interest.

## Publisher’s Note

All claims expressed in this article are solely those of the authors and do not necessarily represent those of their affiliated organizations, or those of the publisher, the editors and the reviewers. Any product that may be evaluated in this article, or claim that may be made by its manufacturer, is not guaranteed or endorsed by the publisher.

## Footnotes

1
https://github.com/jstjohn/SeqPrep

2
https://github.com/najoshi/sickle

3
http://bowtie-bio.sourceforge.net/index.shtml

4
http://deweylab.biostat.wisc.edu/rsem/

5
http://geneontology.org/

6
ftp://ftp.ncbi.nlm.nih.gov/blast/executables/blast+/2.3.0/

7
http://www.genome.jp/kegg/
